# Nonhepatic hyperammonemic encephalopathy complications following bariatric surgery: a case report and review of the literature

**DOI:** 10.1186/s13256-021-02922-2

**Published:** 2021-07-20

**Authors:** Nuphar Vinegrad, Orna Staretz-Chacham, Leonid Barski, Carmi Bartal

**Affiliations:** 1 Internal Medicine, Soroka University Medical Center, Ben Gurion University, Rager Street 151, 8480101 Beer Sheva, Israel; 2 Metabolic Clinic, Soroka University Medical Center, Ben Gurion University, Rager Street 151, 8480101 Beer Sheva, Israel; 3 Neonatlogy Unit, Soroka University Medical Center, Ben Gurion University, Rager Street 151, 8480101 Beer Sheva, Israel

**Keywords:** Encephalopathy, Hyperammonemia, Bariatric surgery, Urea cycle disorder

## Abstract

**Background:**

Hyperammonemic encephalopathy, a rare but fatal condition, is increasingly being reported as a possible complication of bariatric surgery. Here, we present a case of hyperammonemic encephalopathy, focusing on the clinical presentation, diagnostic measures, and our treatment methods, which resulted in a rare favorable outcome, emphasizing the unique role of renal replacement treatment. We also provide a detailed discussion of the mechanism through which hyperammonemia occurs secondarily to bariatric surgery.

**Case presentation:**

A 44-year-old Moroccan Jew woman with a history of obesity presented in the hospital with urea cycle disorder that manifested after bariatric surgery. A rapid diagnostic process, together with conservative treatment with lactulose, nutritional supplementation, dietary protein restriction, and ammonia scavengers did not result in adequate improvement. Therefore, hemofiltration was performed, which yielded a favorable outcome.

**Conclusions:**

The case findings indicate an association between hyperammonemic encephalopathy and bariatric surgery, and support early treatment with ammonia scavengers, as currently accepted. Nevertheless, if rapid improvement is not seen, it is advisable to consider hemodialysis or hemofiltration as early invasive strategies.

## Background

Hyperammonemic encephalopathy is a rare complication associated with bariatric surgery, with high morbidity and mortality rates as high as 50% [1]. In adults, hyperammonemia results from severe liver disease in 90% of cases. Other causes of hyperammonemia are associated with increased ammonia production, such as caused by certain malignant cells during chemotherapy or infections containing urea-producing bacteria. Other causes include a high protein load or increased metabolism, as occurs during seizures, severe exercise, starvation, total parenteral nutrition, or gastrointestinal bleeding. In contrast, hyperammonemia may result from decreased elimination of ammonia as a result of inborn errors of metabolism, congenital portosystemic shunts, or intake of certain drugs that interfere with ammonia elimination metabolism [2].

Elevated ammonia levels are toxic primarily to the brain; consequently, most signs and symptoms of hyperammonemia are neurological. Hyperammonemia in adults can clinically present as acute or chronic hyperammonemia. The symptoms of acute hyperammonemia include vomiting, lethargy, drowsiness, seizures, multiorgan failure, and coma. The range of symptoms of chronic hyperammonemia includes headaches, cognitive deficits, behavioral changes, tremors, ataxia, and seizures [3]. Therefore, early diagnosis and appropriate treatment are crucial to prevent further deterioration in patient condition and mortality. Hyperammonemic encephalopathy after gastric bypass surgery is a newly recognized, potentially fatal syndrome with diverse pathophysiologic mechanisms encompassing genetic and nongenetic causes. Here, we present a case of hyperammonemic encephalopathy, focusing on the clinical presentation, diagnostic measures, and treatment approach, which resulted in a favorable outcome. The mechanism of the development of hyperammonemia secondarily to bariatric surgery is also discussed in detail.

## Case presentation

A 44-year-old Moroccan Jew woman (gravida 4, para 4) was admitted to an internal medicine ward for general weakness and drowsiness, which began 2 days before admission. Her past medical history included morbid obesity and related complications of type 2 diabetes, which were controlled by metformin treatment; dyslipidemia, which was treated with atorvastatin; gastroesophageal reflux disease, which was not being managed with regular treatment; and fatty liver disease with no other known comorbidities. Her family history included untimely deaths of two of her four children, whose symptoms were similar to those in Leigh’s disease; her other two children are alive and healthy. Aiming to lose weight and control additional comorbidities, she underwent gastric band surgery at the age of 30 years. At the age of 38 years, owing to insufficient weight loss, she underwent gastric sleeve bariatric surgery, which resulted in a weight loss of 30 kg; however, she rapidly regained the lost weight. She subsequently remained morbidly obese, with a body mass index of 48.4. She was approved for a third bariatric surgery involving gastric bypass, and was admitted at the age of 44 years, weighing 126 kg. The surgery began with a laparoscopic approach, but, because of bleeding and gastrointestinal leakage, the procedure was converted to an open laparotomy. The procedure included an omega loop gastric bypass and an elective gallbladder resection, but was complicated by heavy postoperative bleeding. During the next several months, she underwent prolonged hospitalization with continuous mechanical ventilation, which required insertion of a tracheostomy tube, several recurring surgeries for peritoneal lavage, and repeated abscess drainage from the internal abdomen and abdominal wall. During this period, the patient also experienced recurrent episodes of sepsis, which required treatment with multiple courses of broad-spectrum antibiotics. Eventually, after 8 months of postoperative hospitalization, she recovered and was transferred to the rehabilitation department.

**Fig. 1:**
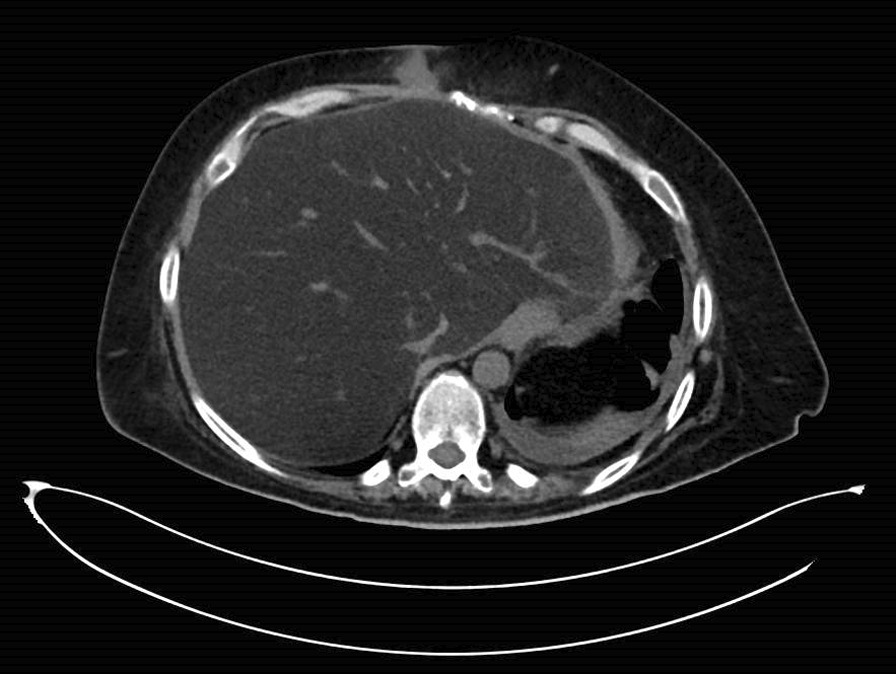
Radiological image. A computed tomography section of the patient, showing diffuse fatty infiltrates in the liver and a small pleural effusion on the left, which was unchanged with respect to previous imaging studies. There is a small surgical site infiltration in the abdominal wall

After 44 days in the rehabilitation department, she gradually developed new symptoms including slow psychomotor response, weakness, worsening peripheral edema, and drowsiness. Her vital signs on admission to the internal medicine department were unremarkable: temperature 36.4 °C, blood pressure 110/72 mmHg, pulse rate 84 beats per minute, respiratory rate 22 breaths per minute, and oxygen saturation on room air 97%. Her weight was 95 kg. Physical examination revealed grade 3 encephalopathy with asterixis, and neurological evaluation revealed hyporeflexia with symmetric weakness and decreased muscle tone. Other findings included peripheral edema grade 3. The surgical wounds in the abdominal wall had closed, and no signs of infection were seen. No tenderness was observed during abdominal palpation. Laboratory blood examinations showed an elevated international normalized ratio (1.6; normal range: below 1.1), severe hypoalbuminemia (1.5 g/dL; normal range: 3.4–5.4 g/dL), and highly elevated blood ammonia (285 µg/dL; normal range: 15–45 µg/dL). Other results included hemoglobin 9.2 g/L, white blood cell count 7200/µL, platelets 234,000/µL, glucose 91 mg/dL, urea 33 mg/dL, creatinine 0.7 mg/dL, sodium 137 mmol/L, potassium 4.3 mmol/L, magnesium 2.4 mmol/L, calcium 7.2 mmol/L, phosphorus 3.4 mmol/L, aspartate aminotransferase (AST) 28 IU/L, alanine aminotransferase (ALT) 31 IU/L, alkaline phosphatase 131 IU/L, gamma glutamyl transpeptidase 34 IU/L, lactate dehydrogenase 413 IU/L, total bilirubin 0.9 mg/dL, creatine phosphokinase 89 IU/L, pH 7.34, bicarbonate 23 mg/dL and PCO_2_ 39.6 mmHg. Her C-reactive protein level was 1.2 mg/L (normal: 0–0.5 mg/L), and her blood ketones were negative. Further laboratory tests revealed normal levels of factor 5 and normal lactate levels (0.6 mg/dL). Imaging studies, including abdominal ultrasound and computerized tomography (CT), did not reveal any signs of cirrhosis; apart from previously known postsurgical changes, only diffuse fatty infiltrates of the liver were found (Fig. 1). Brain CT, including venography and angiography, showed no signs of intracranial pathology, ischemia, or early or late enhancement. Her electroencephalographic findings supported the diagnosis of encephalopathy.

The most likely diagnosis was urea cycle disorder (UCD), as supported by the high ammonia levels, low blood protein levels, and advanced encephalopathy. Imaging studies showed no signs of cirrhosis, and laboratory investigations excluded hepatocellular injury. Hepatic cellular function was preserved, as reflected by standard factor 5 levels. Plasma amino acid analysis revealed a decrease in levels of several amino acid: taurine 17 µM, aspartate 13 µM, citrulline 4 µM, valine 105 µM, leucine 14 µM, tyrosine 25 µM, and histidine 58 µM. Other amino acids were in normal ranges. An acylcarnitine profile showed elevated carnitine with no further abnormalities. The urine organic acid profile revealed markedly elevated 4-hydroxyphenyllactate, but this test was inconclusive because the high levels of antibiotics in the blood might have influenced the results. Karyotyping revealed no irregularities.

Empirical treatments with the ammonia scavenger sodium benzoate, *N*-carbamylglutamate, l-arginine, carnitine, and low-protein total parenteral nutrition were administered in addition to nutritional supplements, which included thiamine, zinc, and vitamins C, B, and E. The patient’s encephalopathy condition continued to deteriorate, with new-onset fever and hemodynamic instability combined with respiratory failure. She was placed on mechanical ventilation, supported by vasopressors, started on a course of broad-spectrum antibiotics, and transferred to the internal intensive care unit, where a second ammonia level test was conducted and indicated a marked increase to 455 µg/dL. Her high ammonia levels were controlled by hemofiltration, in addition to the above treatment, and by administration of supplemental intravenous amino acids. Hemofiltration helped control her ammonia levels, which gradually decreased to baseline levels within 5 days.

A sepsis investigation began after fever and hemodynamic instability further complicated her condition, but no infectious source was found, and all cultures were negative. Total body CT and cardiac echocardiography were performed and yielded normal results.

With the resolution of systemic inflammatory response syndrome (SIRS), our patient was gradually weaned from mechanical ventilation and vasopressors, and her nutrition was steadily shifted from parenteral nutrition to enteral nutrition, and later to oral nutrition. The patient recovered neurologically and regained normal cognitive function. She subsequently developed critical care polyneuropathy, owing to the use of muscle relaxants, and was discharged to undergo rehabilitation in a designated hospital without a further need for nutritional supplements.

After 2 months in the rehabilitation department, she was discharged home. At a 2-year follow up after the hospitalization described above, her hyperammonemia had not recurred, and she had recovered from critical care polyneuropathy. During the follow-up period, no liver disease was found, and her ammonia levels remained stable and in the normal range, as did all other examined blood measurements.

## Discussion and conclusions

**Fig. 2:**
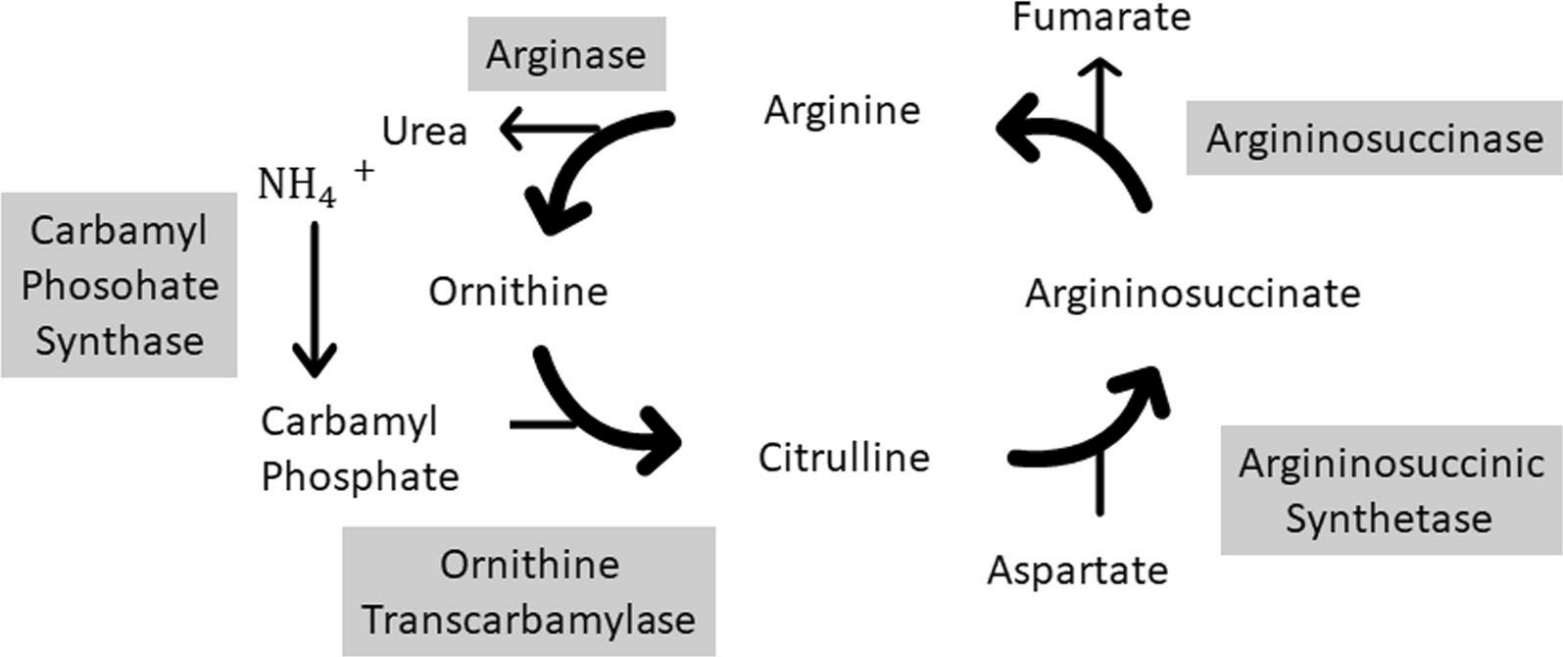
Urea cycle metabolism. Schematic of the biochemical reactions that produce urea from ammonia, referred to as the urea cycle. The image also shows the five key enzymes in the process in gray

Hyperammonemic encephalopathy is a well-recognized condition in infants born with UCD disorders (Fig. 2). This condition has also been reported in adolescents and adults in relation to metabolically stressful events, such as bleeding or surgery, thus causing secondary UCD [4, 5]. With the current improvements in management, children with UCD are able to continue to adulthood. Numerous reports have described hyperammonemia-induced encephalopathy after bariatric surgery as a potential outcome [1, 6–8]. Fenves *et al*. [1] have reviewed data on 20 patients with hyperammonemic syndrome after bariatric surgery, three of whom were diagnosed with UCD. However, for the remainder of the patients, the reason for the link between hyperammonemia syndrome and surgery remained unclear. Several studies have aimed to explain this connection. One possible explanation is the unmasking of an already-existing urea cycle enzyme deficiency that did not present earlier [7, 8], given that several of these disorders have a wide spectrum of symptoms and age presentation [9]. A high nitrogen load resulting from rapid weight loss after bariatric surgery accompanied by operation-related hyperinsulinemia and zinc deficiency can downregulate the expression of urea cycle enzymes and interfere with ornithine transcarbamylase function [10, 11]. Additionally, gastric bypass has been reported to interfere with citrulline synthesis in the intestinal wall, thereby depleting urea cycle substrates and subsequently inhibiting the urea cycle function [12]. Another possible explanation linking bariatric surgery and hyperammonemia is gut microbiome alterations favoring urealytic strains that may contribute to increased ammonia production [1].

**Fig. 3:**
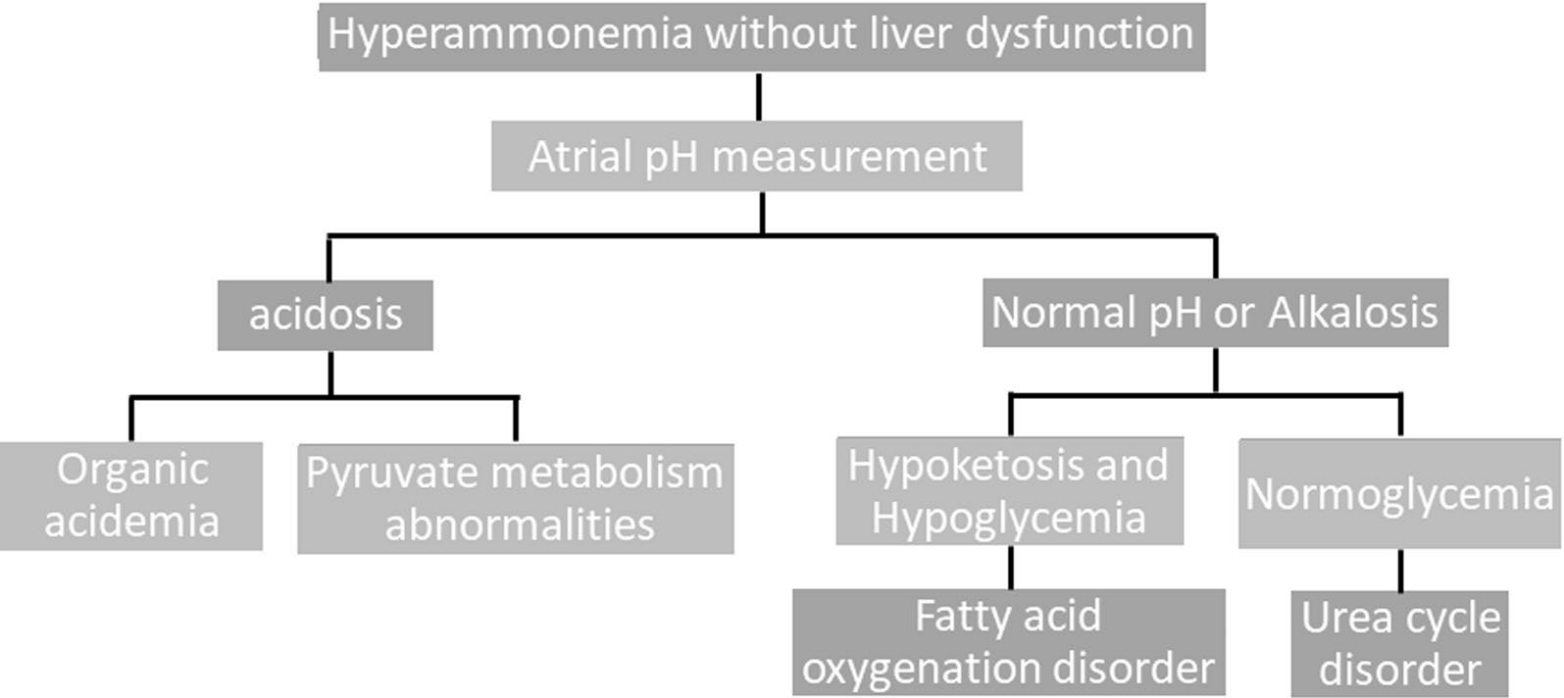
Diagnostic algorithm for nonhepatic hyperammonemia. Diagnostic algorithm, showing the first steps for diagnosing the cause of nonhepatic hyperammonemia (based on studies by Ruby Upadhyay, Thomas P. Bleck, and Katharina M. Busl [10]; Jamie Nicole LaBuzetta, Phil Jay Z. Yao, Daniel L. Bourque, and JustinZivin [13]; and Alison S. Clay and Bryan E. Hainline [14])

Several studies have presented a workup algorithm for nonhepatic hyperammonemia [10, 13, 14] that uses three steps. The first step involves measuring the arterial pH; if there is no acidosis, the next step is the measurement of blood ketone and glucose levels (Fig.3). Normal pH or alkalosis without ketosis or hypoglycemia leads to a suspicion of UCD, as with our patient.

**Fig. 4:**
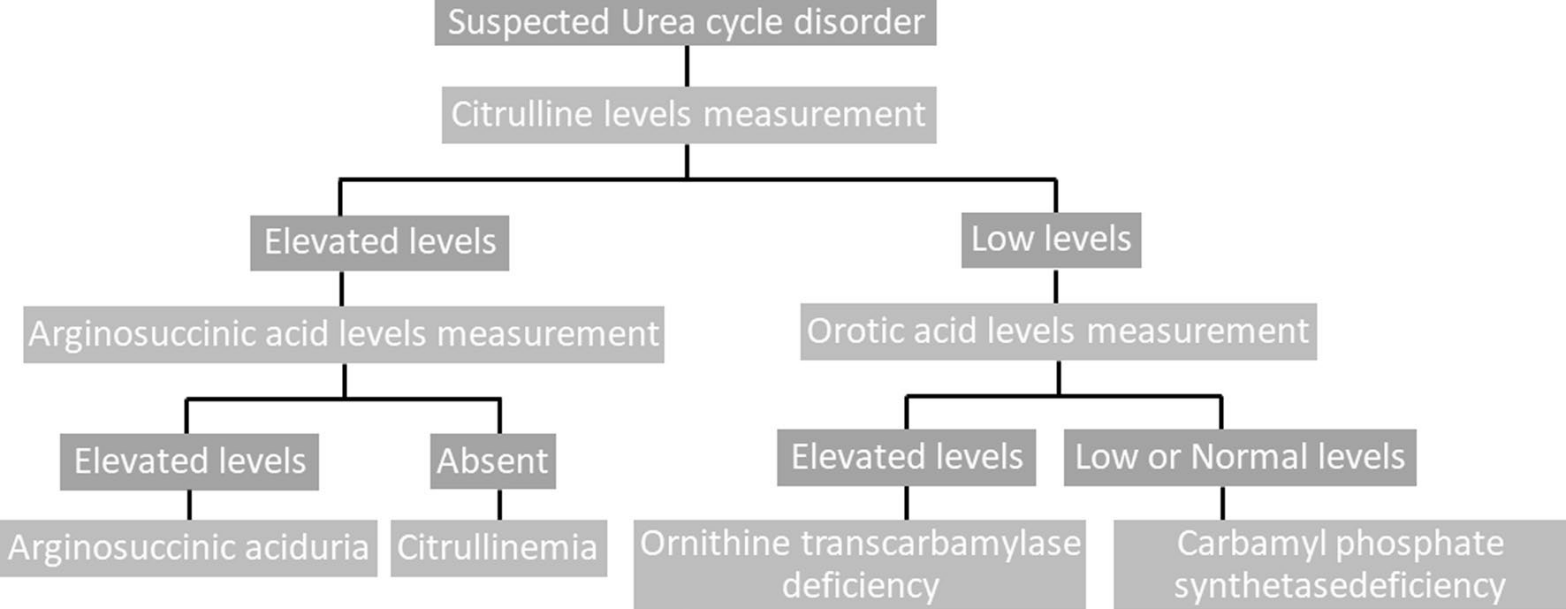
Diagnostic workup for a suspected urea cycle disorder, on the basis of plasma amino acid analysis. Diagnostic workup showing the steps for diagnosing the cause of a suspected urea cycle disorder, on the basis of plasma amino acid analysis (based on studies by Ruby Upadhyay, Thomas P. Bleck, and Katharina M. Busl [10]; Jamie Nicole LaBuzetta, Phil Jay Z. Yao, Daniel L. Bourque, and JustinZivin [13]; and Alison S. Clay and Bryan E. Hainline [14])

Further evaluation (Fig. 4) includes measurement of citrulline, argininosuccinic acid and orotic acid levels. Our patient had low citrulline levels but normal levels of orotic acid, thus ruling out the possibility of ornithine transcarbamylase deficiency.

**Fig. 5:**
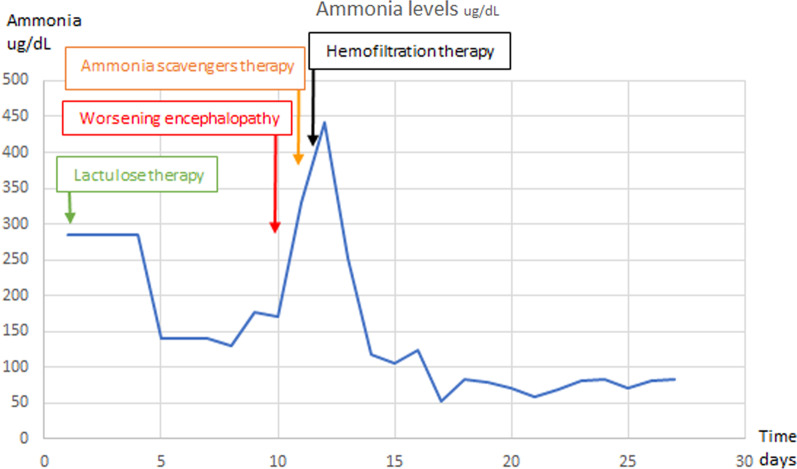
Ammonia levels and treatment trials. A graphic depiction of the patient’s ammonia levels on each day, indicated by the blue line. The different treatment methods and the days on which they began are shown above the blue line

After hyperammonemic encephalopathy is suspected as a potential diagnosis, regardless of its relationship with bariatric surgery, rapid treatment should begin before the final diagnosis, owing to the high rate of morbidity and mortality [14]. Sodium benzoate, sodium phenylbutyrate, and arginine are ammonia scavengers, which facilitate nitrogen conversion to non-urea products that are readily excreted and improve cognitive impairment when they are combined with additional dietary protein restriction [10, 14]. Under these treatment regimens together with lactulose therapy, which started as soon as her symptoms presented, our patient’s SIRS condition worsened, as supported by her high-grade fever and rapid respiratory rate, which developed gradually from the time of presentation and eventually deteriorated to hemodynamic and respiratory failure, all without an apparent infectious cause. Because our patient did not show any improvement, an additional hemofiltration treatment was performed. Several studies have shown the benefit of treatment with hemodialysis or hemofiltration [1, 14]. Our patient showed a rapid response, including a sharp decline in ammonia levels under hemofiltration and reversal of the SIRS symptoms (Fig. 5), thus suggesting that hemofiltration led to the favorable outcome in this case.

In conclusion, our case findings contribute to the understanding that hyperammonemic encephalopathy may be a bariatric surgery complication that can be fatal because of severe hyperammonemia accompanied by life-threatening SIRS symptoms. Although this case report is a rare single patient description, given the potentially fatal outcomes of treatment failure and the cumulative reports of the possible advantages of hemofiltration [1, 14], our findings underscore the importance of early and aggressive treatment. In cases in which conservative treatment with lactulose, nutritional supplements, dietary protein restriction, and ammonia scavengers do not produce rapid improvement, early hemodialysis or hemofiltration therapy should be provided to aid in achieving favorable outcomes.

## Data Availability

The data that support the findings of this study are available from the Camilion information system at Soroka University Medical Center, but restrictions apply to the availability of these data, which were used under license for the current study and therefore are not publicly available. However, data are available from the authors upon reasonable request with the permission of Soroka University Medical Center.

## Abbreviations

CT: Computed tomography
SIRS: Systemic inflammatory response syndrome
UCD: Urea cycle disorder
